# Circulating levels of MOTS-c in patients with breast cancer treated with metformin

**DOI:** 10.18632/aging.204423

**Published:** 2022-12-06

**Authors:** Elisabet Cuyàs, Sara Verdura, Begoña Martin-Castillo, Javier A. Menendez, METTEN study group

**Affiliations:** 1 Metabolism and Cancer Group, Program Against Cancer Therapeutic Resistance (ProCURE), Catalan Institute of Oncology, Girona 17005, Spain; 2 Girona Biomedical Research Institute, Girona 17190, Spain; 3 Clinical Research Unit, Catalan Institute of Oncology, Girona 17005, Spain

**Keywords:** MOTS-c, metformin, breast cancer

## Abstract

The mitokine MOTS-c is a mitochondrially-encoded “exercise-mimetic peptide” expressed in multiple tissues, particularly skeletal muscles, which can be detected as a circulating hormone in the blood. MOTS-c mechanisms of action (MoA) involve insulin sensitization, enhanced glucose utilization, suppression of mitochondrial respiration, and targeting of the folate-AICAR-AMPK pathway. Although MOTS-c MoA largely overlap those of the anti-diabetic biguanide metformin, the putative regulatory actions of metformin on MOTS-c have not yet been evaluated in detail. Here, we measured circulating MOTS-c in paired baseline and post-treatment sera obtained from HER2-positive breast cancer patients randomized to receive either metformin combined with neoadjuvant chemotherapy and trastuzumab or an equivalent regimen without metformin. We failed to find any significant alteration of circulating MOTS-c –as measured using the commercially available competitive ELISA CEX132Hu– in response to 24 weeks of a neoadjuvant chemotherapy/trastuzumab regimen with or without daily metformin. Changes in circulating MOTS-c levels failed to reach statistical significance when comparing patients achieving pathological complete response (pCR), irrespective of metformin treatment. The inability of metformin to target skeletal muscle, the major tissue for MOTS-c production and secretion, might limit its regulatory effects on circulating MOTS-c. Further studies are needed to definitely elucidate the nature of the interaction between metformin and MOTS-c in cancer and non-cancer patients.

## INTRODUCTION

Mitochondrial open reading frame of the 12S ribosomal RNA type-c (MOTS-c) is a mitochondrially-encoded 16-amino-acid biopeptide that functions as an exercise-induced regulator of metabolic homeostasis [1, 2] and a modulator of obesity-, diet-, and aging-dependent metabolic function by acting as a systemic, endocrine-acting mitokine [3, 4]. MOTS-c dynamically translocates to the nucleus in response to metabolic stress to directly regulate the expression of a broad spectrum of genes, including antioxidant response element-containing target genes [5]. By targeting folate-dependent *de novo* purine biosynthesis, MOTS-c boosts the levels of the endogenous AMP analog 5-aminomidazole-4-carboxamide ribonucleotide (AICAR), which in turn activates the master energy sensor AMP-activated protein kinase (AMPK). Skeletal muscle cell-targeted involvement of the folate-AICAR-AMPK pathway constitutes the mechanistic basis of MOTS-c as an endogenous “exercise mimetic”, which can stimulate glucose utilization and fat oxidation and suppress inflammation [4].

Exercise interventions show promise as effective adjunct strategies to prevent and/or attenuate chemotherapy-associated toxicity (e.g., cardiotoxicity and cardiopulmonary dysfunction) in patients with early-stage breast cancer (BC) [6, 7]. Exploratory studies have demonstrated that exercise interventions might also modulate host- and tumor-related pathways in patients on standard chemotherapy [8]. Indeed, several ongoing and planned interventional studies (e.g., Neo-ACT NCT05184582, Neo-Train NCT04623554) have been designed to examine whether physical exercise interventions during the neoadjuvant chemotherapy period can bolster treatment efficacy [9, 10]. Pharmacological therapeutics that partially mimic the systemic impact of exercise have also been proposed for those cancer patients for whom exercise training may not be an option [11–13]. One putative exercise mimetic, metformin, shares many mechanistic features with MOTS-c, including: (a) insulin sensitization, (b) enhancing glucose utilization, (c) suppressing mitochondrial respiration, and (d) targeting the folate-AICAR-AMPK pathway [4, 14–16]. To date, however, no study has examined whether metformin can influence the expression of MOTS-c in cancer patients. Here, we explored the impact of metformin on circulating MOTS-c levels in the METTEN study (EudraCT number 2011-000490-30), a phase 2 clinical trial of women with HER2-positive BC randomized to receive either metformin (850 mg twice daily) for 24 weeks concurrently with 12 cycles of weekly paclitaxel plus trastuzumab, followed by four cycles of 3-weekly FE75C plus trastuzumab (arm A), or an equivalent regimen without metformin (arm B), before surgery [17].

**Figure 1:**
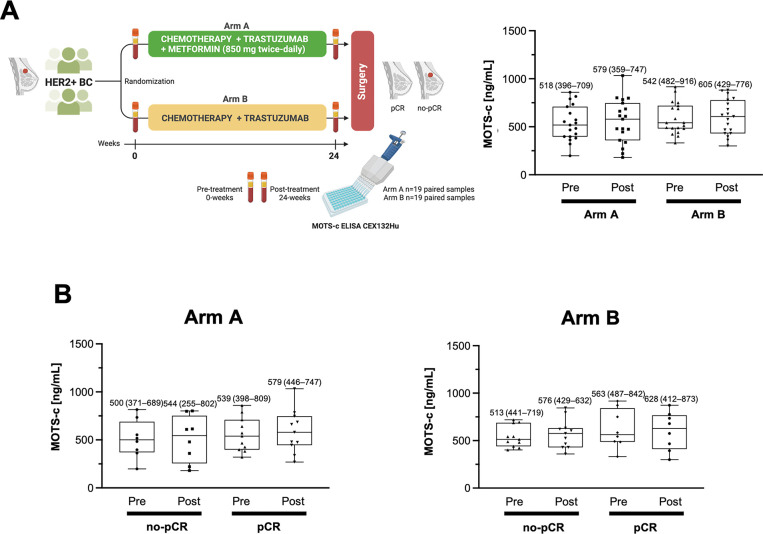
**Circulating levels of MOTS-c in patients with HER2+ breast cancer treated with neoadjuvant metformin.** (**A**) Left. METTEN study design. Circulating MOTS-c levels were determined through blood draws obtained at pre- (0 weeks) and post- (24 weeks) treatment using a commercial ELISA kit (CloudClone Corp., Wuhan, China; Catalog No. CEX132Hu). Right. Box plot (median, 25%–75% quartiles and minimal and maximal values) of the pre- and post-treatment distribution of circulating MOTS-c in women randomized to arms A (metformin-containing) and B (without metformin). (**B**) Box plot (median, 25%–75% quartiles and minimal and maximal values) of the pre- and post-treatment distribution of circulating MOTS-c in non-pCR and pCR groups. No between-group comparisons reached statistical significance in A and B.

The present study was conducted with paired baseline and post-treatment serum samples collected from 38 patients (n=19 in each arm) belonging to the intention-to-treat population of the METTEN trial (Figure 1A, left), which included randomly assigned patients receiving at least one dose of study medication. All samples were evaluated in parallel for circulating MOTS-c using a commercially available competitive ELISA (CEX132Hu; CloudClone Corp., Wuhan, China) [18, 19]. Within- and between-group data were assessed by paired t-test and *post hoc* Tukey multiple comparison tests on repeated measures ANOVA. No statistically-significant differences were found between the pre- and post-levels of circulating MOTS-c irrespective of the treatment arm (Figure 1A, right). Similarly, changes in circulating MOTS-c levels failed to reach statistical significance when comparing patients achieving pathological complete response (pCR), defined as the absence of invasive tumor cells in post-neoadjuvant therapy surgical histopathology of the complete resected breast specimen, including sample regional lymph nodes [17], and patients with non-pCR, irrespective of the treatment arm (Figure 1B).

Our findings indicate that metformin does not operate as an exercise mimetic to augment the circulating levels of MOTS-c in patients with BC treated with neoadjuvant therapy. It is possible that the lack of effect relates to different target tissues of metformin and MOTS-c. We have recently learned that an expansion of the skeletal muscle-derived MOTS-c protein pool occurs concomitantly with increases in mitochondrial DNA [20], which might suggest that circulating MOTS-c could operate as a surrogate marker of phenotypic and functional shifts of mitochondrial networks [21]. Metformin is known to activate AMPK when targeting the liver, kidney, and intestine but not skeletal muscle [22] –the major tissue for MOTS-c production and secretion–, which might prevent any regulatory effect on circulating MOTS-c. Moreover, we are accumulating evidence that metformin does not enhance (and instead dampens) the beneficial strength gains and muscle activation in response to exercise training in healthy elderly people [23–26]. The mechanisms of MOTS-c production, secretion, distribution, and metabolism in the human body remain to be fully elucidated. Likewise, the extent of involvement of various tissue targets and/or the effects of metformin on skeletal muscle metabolism and how they determine the pharmacodynamics and endogenous serum levels of MOTS-c in patients with BC await evaluation in future studies. One should acknowledge that plasma and muscle MOTS-c show opposing responses to aging in older men, thereby suggesting that the primary source of circulating MOTS-c is not skeletal muscle or the pharmacokinetics of MOTS-c changes with age [27]. Similarly, the ability of muscle cells to release MOTS-c can be impaired due to changes in the export process and/or to the exhausted capacity of muscle (or hepatic) cells to tolerate or adapt to systemic metabolic stress occurring in cancer patients. Therefore, we cannot exclude the possibility that serum circulating MOTS-c and muscle MOTS-c can be differentially regulated by metformin, as aging does [27].

There are several limitations to this study. Endogenous levels of circulating MOTS-c have been shown to vary significantly (from 154 pg/mL to 584 ng/mL) depending on the assay method used [5, 18, 19, 28–30]. In our series of patients, the endogenous serum levels of MOTS-c ranged from 181 ng/mL to 1033 ng/mL. Overall, these findings would strongly suggest that the immunoreactive species of circulating MOTS-c detected using different kits are not identical. Our previous analysis confirmed that treatment of non-diabetic patients with HER2+ BC with oral metformin (850 mg twice daily) for 24 weeks produced blood levels of circulating metformin of ~7 μmol/L, equivalent to those generally achieved in diabetic patients with the usual clinical doses and schedule [17]. The exercise-induced augmentation of circulating MOTS-c in young subjects was found to return to baseline after only 4 hours of resting [4]. As we measured circulating MOTS-c in blood that was not strictly timed in relation to the last preceding oral dose of metformin [17], our data need to be viewed cautiously in terms of association between metformin treatment, achieved serum concentration of MOTS-c, and probability of pCR in BC patients. Moreover, the METTEN trial was conducted in patients with the HER2+ subtype of breast cancer, which leaves open the question of whether the circulating levels of MOTS-c and/or the regulatory activity of metformin on MOTS-c might be different in patients with other BC subtypes, such as luminal A, HER2-negative luminal B or triple negative [31]. Nonetheless, this is a retrospective study in a small sample size for which the evaluation of MOTS-c was not part of the original study design. Care should therefore be taken in interpreting and generalizing these findings. With an ever-growing recognition of the role of MOTS-c on age-related diseases including diabetes, obesity, osteoporosis, cardiovascular, and neurodegenerative diseases [32, 33], further studies are needed to elucidate the nature of the interaction between metformin and MOTS-c in cancer and non-cancer patients.
